# Serum Phosphorus and Albumin in Patients Undergoing Peritoneal Dialysis: Interaction and Association With Mortality

**DOI:** 10.3389/fmed.2021.760394

**Published:** 2021-12-01

**Authors:** Naya Huang, Huiyan Li, Li Fan, Qian Zhou, Dongying Fu, Lin Guo, Chunyan Yi, Xueqing Yu, Haiping Mao

**Affiliations:** ^1^Department of Nephrology, The First Affiliated Hospital, Sun Yat-sen University, Key Laboratory of Nephrology, Ministry of Health of China, Guangdong Provincial Key Laboratory of Nephrology, Guangzhou, China; ^2^Department of Medical Statistics, Clinical Trials Unit, The First Affiliated Hospital, Sun Yat-sen University, Guangzhou, China; ^3^Department of Nephrology, Guangdong Provincial People's Hospital, Guangzhou, China

**Keywords:** serum phosphorus, albumin, peritoneal dialysis, interaction, mortality

## Abstract

Hyperphosphatemia and hypoalbuminemia confer worse clinical outcomes, whether these risk factors interact to predispose to mortality is unclear. In this prospective cohort study, 2,118 patients undergoing incident continuous ambulatory peritoneal dialysis (CAPD) were enrolled and categorized into four groups based on the changing point regarding mortality at 1.5 mmol/L for serum phosphorus and 35 g/L for serum albumin. Risks of all-cause and cardiovascular mortality were examined independently and interactively in overall and subgroups. There was no association between serum phosphorus with all-cause and cardiovascular mortality, but significant interactions (*p* = 0.02) between phosphorus and albumin existed in overall population. Patients in subgroup with high phosphorus and low albumin were at greater risk of all-cause (HR 1.95, 95%CI 1.27–2.98, *p* = 0.002) but not cardiovascular mortality (HR 0.37, 95%CI 0.10–1.33, *p* = 0.13), as compared to those with low phosphorus and high albumin. In contrast, patients with both low parameters had a higher risk of all-cause (HR 1.75, 95%CI 1.22–2.50, *p* = *0.002*) and cardiovascular mortality (HR 1.92, 95%CI 1.07–3.45, *p* = *0.03*). Notably, an elevated risk of both all-cause and cardiovascular mortality was observed in those with low serum albumin, irrespective of phosphorus levels, suggesting low albumin may be useful to identify a higher-risk subgroup of patients undergoing CAPD with different serum phosphorus levels.

## Introduction

Hyperphosphatemia is present in almost 40% patients undergoing dialysis (1–3)_._ Previous studies indicate that a higher serum phosphorus level is associated with an increased risk of cardiovascular events, cardiovascular and all-cause mortality in patients undergoing pre-dialysis and dialysis (4–12). Restriction of dietary phosphorus intake is recommended as one of the key strategies in clinical management of hyperphosphatemia (13). Although foods with high protein are major source of phosphorus, control of dietary phosphorus by reducing protein intake has been linked to hypoalbuminemia (13–17). Because hypoalbuminemia is also a strong predictor of adverse clinical outcomes in patients undergoing dialysis (1, 5, 7, 11, 18–22), how to balance the relation between serum phosphorus and albumin and their interactive impacts on mortality risk remains a clinical conundrum.

It has been demonstrated that restricting dietary protein intake to reduce serum phosphorus may cause greater mortality in patients undergoing dialysis and outweigh the benefit of controlled phosphorus (14, 16). A previous study on hemodialysis (HD) has shown that subjects whose protein intake decreased had higher mortality compared to those whose protein intake rose over 6 months, regardless of serum phosphorus levels (16). Moreover, Zitt et al. (20) proposed that albumin and phosphorus interact with each other in their associations with mortality in patients undergoing HD, and concurrent low phosphorus and high albumin was associated with the lowest risk of mortality in patients undergoing HD, but this relationship was not found in patients treated by peritoneal dialysis (PD) possibly because of small study population (*n* = 38). Unlike HD, peritoneal protein loss *via* peritoneal effluent has been recognized as a major disadvantage in PD; patients on PD have lower serum albumin than those undergoing HD (23–26). Meanwhile, Mehrotra et al. (27) has found that serum albumin levels as a risk factor for mortality were shifted to a lower range in PD, as compared to HD. Hence, the interplay between phosphorus and albumin in patients on HD could not be interpreted in patients on PD. Furthermore, evidence from longitudinal studies regarding PD population is scarce. Therefore, we intended to evaluate serum phosphorus–albumin interaction and their associations with all-cause and cardiovascular mortality among patients treated by continuous ambulatory peritoneal dialysis (CAPD).

## Materials and Methods

### Study Population

This was a single center, prospective, observational cohort study. All patients on incident CAPD at The First Affiliated Hospital, Sun Yat-Sen University were recruited from January 1, 2006 to December 31, 2016. Eligible participants were older than 18 years and had been on CAPD more than 3 months. Patients who were transferred from maintenance HD or failed kidney transplantation, had malignant disease, or refused to given written consent were excluded. All patients were treated with glucose-based Dianeal PD solution (Baxter Healthcare Ltd., Guangzhou, China). The study was approved by the Clinical Research Ethics Committee of The First Affiliated Hospital of Sun Yat-Sen University, and all patients provided written informed consent before enrollment.

### Data Collection

Baseline demographic, clinical, and laboratory data were collected within 3 months after CAPD commencement and obtained from our database. Demographic and clinical characteristics included age, sex, body mass index (BMI), blood pressure, primary cause of kidney failure, and concomitant disease. Cardiovascular disease was defined as the presence of ischemic heart disease, congestive heart failure, cerebrovascular disease, or peripheral vascular disease. Laboratory variables included hemoglobin, serum albumin, serum phosphate, calcium, intact parathyroid hormone (iPTH), alkaline phosphatase (ALP), creatinine cholesterol, triglyceride, high-density lipoprotein (HDL), low-density lipoprotein (LDL), high-sensitivity C-reactive protein (hs-CRP), and residual renal function (RRF, defined as mean value of 24-h urinary urea and creatinine clearance). Normalized protein catabolic rate (nPCR), total Cr clearance, and total Kt/*V*_urea_ were calculated using the PD Adequest software (Baxter Healthcare Corporation, Deerfield, IL, USA).

Medications including phosphorus binders, calcitriol, cinacalcet, and α-Ketoacid were recorded. Patients were asked to undergo follow-up visits at the hospital every 3 months and subjected to a routine blood test, including serum albumin and serum phosphate.

### Outcomes

Our primary outcomes were all-cause and cardiovascular mortalities. Cardiovascular mortality was defined as death due to acute myocardial infarction, ischemic heart disease, cardiomyopathy, fatal arrhythmia, cardiac arrest, congestive heart failure, cerebrovascular accident (including intracranial hemorrhage and subdural hematoma, cerebral infarction), and peripheral vascular disease (28). All the patients were followed up until death, transferred to HD, kidney transplantation, transferred to other centers, loss of follow-up, or until December 31st, 2020.

### Statistical Analysis

Results of continuous variables were expressed as mean ± SD for normal distribution and median (interquartile range, IQR) for skewed distribution. For categorical variables, the results were expressed as frequencies and percentages. Pairwise deletion (available case analysis) was used to address the missing data. Restrict cubic splines with five knots at the 5th, 35th, 50th, 65th, and 95th centiles were used to test the linearity of serum phosphorus and albumin and their relationships with all-cause and cardiovascular mortality. A two-line piecewise linear model was next fitted by trying all possible values to approach the change point with highest likelihood. Patients were categorized into four groups based on the changing point regarding mortality at 1.5 mmol/L for serum phosphorus and 35 g/L for serum albumin according to restricted cubic splines and subsequent piecewise-linear models, and subgroups were categorized as follows: low phosphorus and high albumin group with phosphorus < 1.5 mmol/L and albumin ≥35 g/L; low phosphorus and low albumin group with phosphorus < 1.5 mmol/L and albumin < 35 g/L; high phosphorus and high albumin group with phosphorus ≥1.5 mmol/L and albumin ≥35 g/L; and high phosphorus and low albumin group with phosphorus ≥1.5 mmol/L and albumin < 35 g/L. Further comparisons between the subgroups were analyzed using Kaplan–Meier curves and Cox regression models. Unadjusted and adjusted Cox proportional hazard regression models were used to evaluate the associations and interactions of serum phosphorus and albumin with all-cause and cardiovascular mortality. All statistical analyses were performed using the IBM SPSS software, version 22.0 (IBM Corp., Armonk, NY, USA) and R 4.0.2 (https://www.r-project.org/). The *p* < 0.05 was considered statistically significant.

### Baseline Characteristics

**Figure 1:**
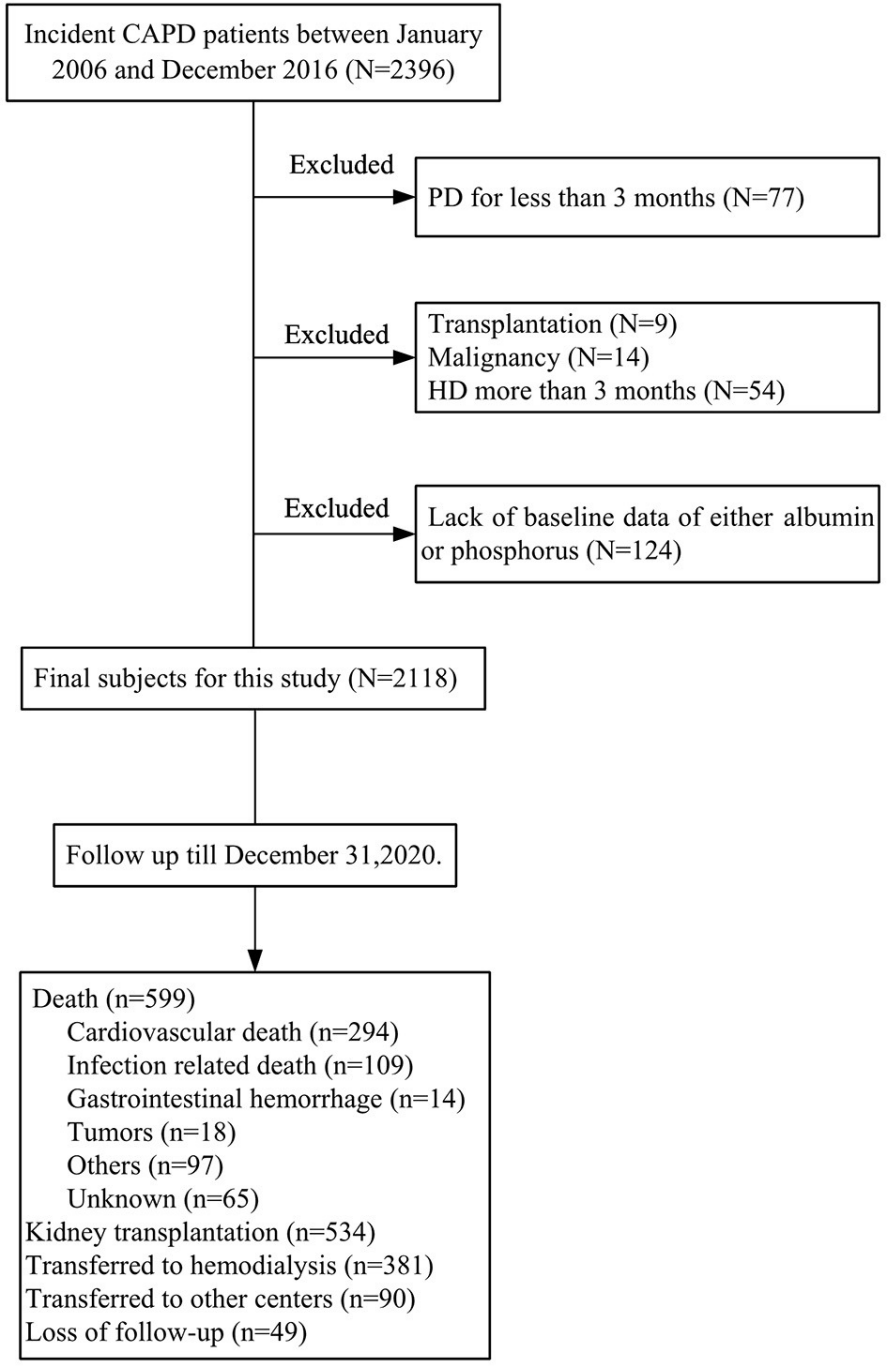
Subjects' inclusion flowchart.

**Figure 2:**
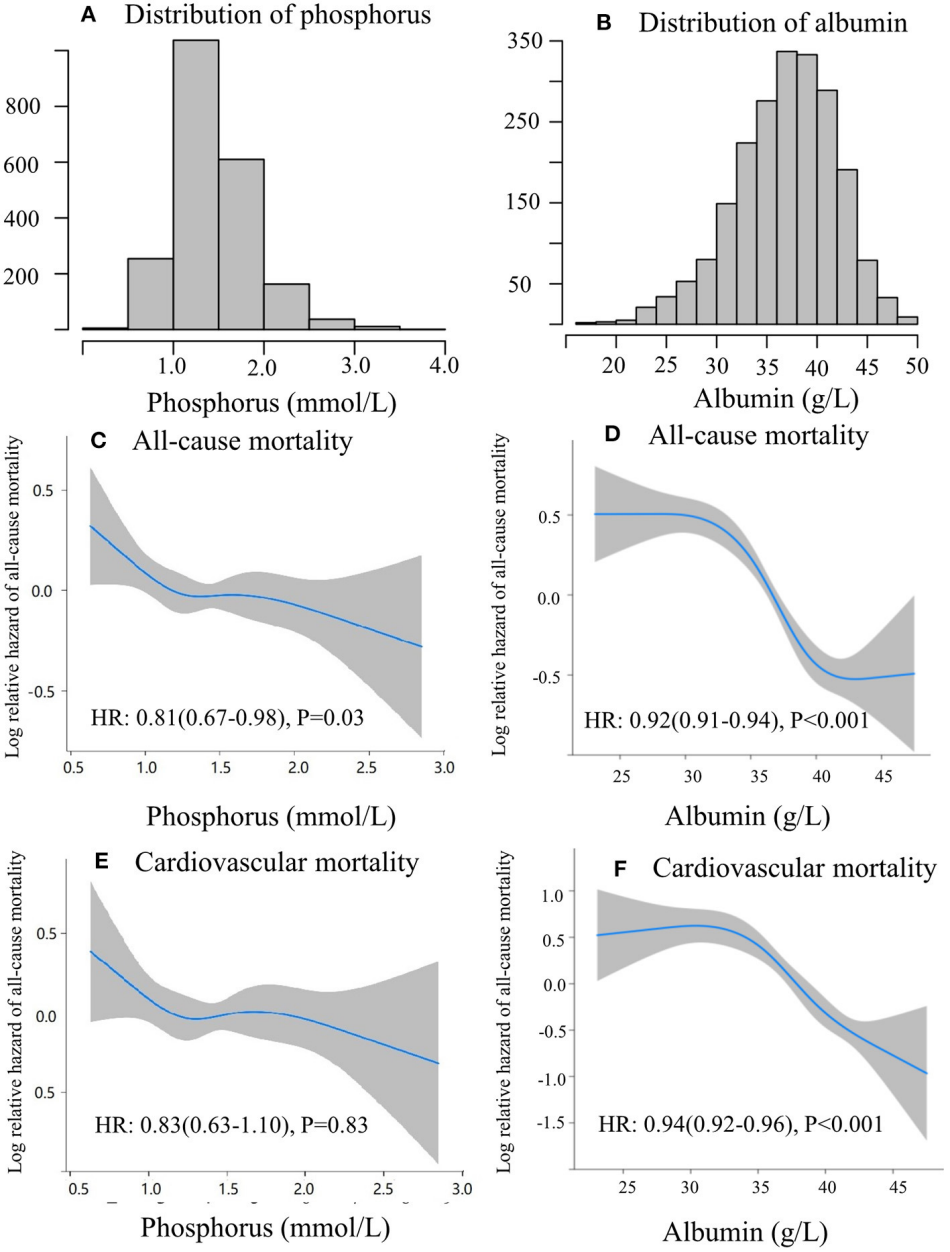
Linear and non-linear correlation between serum phosphorus and albumin with mortality. The distribution of serum phosphorus **(A)**, and albumin **(B)**. Restricted cubic spline of phosphorus **(C)**, albumin **(D)** and all-cause mortality; restricted cubic spline of phosphorus **(E)**, albumin **(F)**, and cardiovascular mortality.

The screening flowchart of patients is shown in Figure 1. Between January 2006 and December 2016, a total of 2,118 patients who underwent consecutive CAPD were enrolled in the present study. Baseline characteristics, including demographic, clinical, laboratory values, and medications, are summarized in Table 1. Mean age of the study cohort was 48 ± 16 years, 59% of them were male, 25% had diabetes, and 22% had a history of cardiovascular disease. The baseline serum phosphorus level was 1.45 ± 0.43 mmol/L, serum albumin level was 37.0 ± 5.08 g/L, and nPCR was 0.9 ± 0.3 g/kg/day. Median level of RRF was 4 (2–17) ml/min/1.73 m^2^. Distributions of serum phosphorus and albumin are shown in Figures 2A,B. For medications, 849 (40%) patients were treated with phosphate binders, 635 (30%) received calcitriol, and 777 (37%) were supplemented with α-ketoacid.

**Table 1:** Baseline characteristics of study cohort.

**Variable**	**Total (*n* = 2,118)**
Age (years)	48 ± 16
Male gender	1,255 (59%)
**Primary kidney disease**	
Chronic glomerulonephritis	1,252 (59%)
Diabetic nephropathy	347 (16%)
Hypertensive nephrosclerosis	284 (13%)
Others	232 (11%)
**Comorbid conditions**	
History of cardiovascular disease	455 (22%)
History of DM	522 (25%)
BMI (kg/m^2^)	21.6 ± 3.2
SBP (mmHg)	139 ± 18
DBP (mmHg)	85 ± 24
Hemoglobin (g/L)	106 ± 23
Serum albumin (g/L)	37.0 ± 5.08
Calcium (mmol/L)	2.25 ± 0.22
Serum phosphorus (mmol/L)	1.45 ± 0.43
iPTH (pg/mL)	235 (114, 406)
Cholesterol (mmol/L)	4.9 (4.1–5.7)
Triglyceride (mmol/L)	1.72 ± 1.41
LDL-C (mmol/L)	2.95 ± 1.03
HDL-C(mmol/L)	1.20 ± 0.55
hs-CRP (mg/L)	1.6 (0.5–5.1)
nPCR (g/kg/d)	0.9 ± 0.3
RRF (ml/min/1.73 m^2^)	4 (2-17)
**Baseline medications**	
Phosphorus binders	849 (40%)
Calcitriol	635 (30%)
α-Ketoacid	777(37%)

*The results are expressed as means ± SD for normal distributed continuous variables, median (25th percentile and 75th percentile) for skew continuous variables or number (%) for categorical variables.*

*BMI, body mass index; DBP, diastolic blood pressure; DM, diabetes mellitus; HDL-C, high-density lipoprotein cholesterol; hs-CRP, high sensitive C-reactive protein; iPTH, intact parathyroid hormone; LDL-C, low-density lipoprotein cholesterol; nPCR, normalized protein catabolic rate; RRF, residual renal function; SBP, systolic blood pressure*.

### Independent and Interactive Associations of Serum Phosphorus and Albumin With Mortality

During a median follow-up of 49 months, 599 (28%) patients died. Among these patients, 294 (49%) were caused by cardiovascular disease and 109 (18%) by infectious disease. The clinical outcomes of the study patients were listed in Table 2. The mortality predictability of serum phosphorus and albumin was assessed separately and interactively (Table 3). In the multivariate Cox regression models, higher serum albumin level was associated with decreased risk of all-cause (HR 0.93, 95%CI 0.89–0.98, *p* = 0.003) and cardiovascular mortality (HR 0.95, 95%CI 0.91–0.99, *p* = 0.03), whereas higher serum phosphorus level was not associated with all-cause (HR 0.71, 95%CI 0.45–1.14, *p* = 0.16) or cardiovascular mortality (HR 0.75, 95%CI 0.49–1.17, *p* = 0.18), after adjustment for age, gender, history of diabetes and cardiovascular disease, serum levels of hemoglobin, hs-CRP, iPTH, blood pressure, total cholesterol, TG, LDL-c, RRF, and uses of phosphate binders, calcitriol, or α-ketoacid. Moreover, significant interactions were detected between phosphorus and albumin on mortality after adjusting for confounding variables (*p-*interaction = 0.02 for both all-cause and cardiovascular mortality). In addition, we observed the non-linear associations of serum phosphorus and albumin with all-cause and cardiovascular mortality (*p* < 0.001 for both) in the restricted cubic splines analysis (Figure 2), suggesting serum phosphorus and albumin might modify each other in their relations to outcomes.

**Table 2:** Outcomes of the study cohort.

**Variables**	***n*** **(%)**
Follow-up (months)	49 (20, 78)
**Outcomes**	
Deaths	599 (28%)
Cardiovascular death	294 (49%)
Infection	109 (18%)
Gastrointestinal hemorrhage	14 (2%)
Tumors	18 (3%)
Others	97 (16%)
Unknown	65 (11%)
Kidney transplantation	534 (25%)
Transferred to hemodialysis	381 (18%)
Transferred to other centers	90 (4%)

*Values are median (25–75%) or n (%)*.

**Table 3:** Interaction and association of albumin and phosphorus with all-cause mortality and cardiovascular mortality.

	**Model 1**		**Model 2**		**Model 3**	
	**HR (95% CI)**	***p-*** **value**	**HR (95% CI)**	***p-*** **value**	**HR (95% CI)**	***p-*** **value**
**All-cause mortality**						
Phosphorus (mmol/L)	0.81 (0.67–0.98)	**0.03**	1.07 (0.89–1.29)	0.48	0.71 (0.45–1.14)	0.16
Albumin (g/L)	0.92 (0.91–0.94)	** <0.001**	0.96 (0.94–0.97)	** <0.001**	0.93 (0.89–0.98)	**0.003**
Interaction		** <0.001**		**0.03**		**0.02**
**Cardiovascular mortality**						
Phosphorus (mmol/L)	0.83 (0.63–1.10)	0.83	1.11 (0.85–1.45)	0.45	0.75 (0.49–1.14)	0.18
Albumin (g/L)	0.94 (0.92–0.96)	** <0.001**	0.97 (0.95–0.99)	**0.01**	0.95 (0.91–0.99)	**0.03**
Interaction		**0.001**		0.32		**0.02**

*HR, hazard ratio.*

^*^Bold indicates significance at p < 0.05.

*Model 1 unadjusted.*

*Model 2 adjusted for: age and gender.*

*Model 3 adjusted: model 2+ history of diabetes and cardiovascular disease, serum levels of hemoglobin, high sensitive C-reactive protein, intact parathyroid hormone, systolic blood pressure, diastolic blood pressure, total cholesterol, triglyceride, low-density lipoprotein cholesterol, residual renal function, and uses of phosphate binders, calcitriol, and α-ketoacid*.

### Combined Effect of Serum Phosphorus and Albumin on Mortality

By trying all the possible values for the change points with highest likelihoods in the non-linearity models in restricted cubic splines, two-line piecewise-linear models were used. Since 1.5 mmol/L for serum phosphorus and 35 g/L for serum albumin had the highest likelihood, those values were chosen for a categorical split. Thereby, patients were categorized into four groups based on the four-level joint phosphorus/albumin concentrations, which were described in detail in materials and methods section.

**Figure 3:**
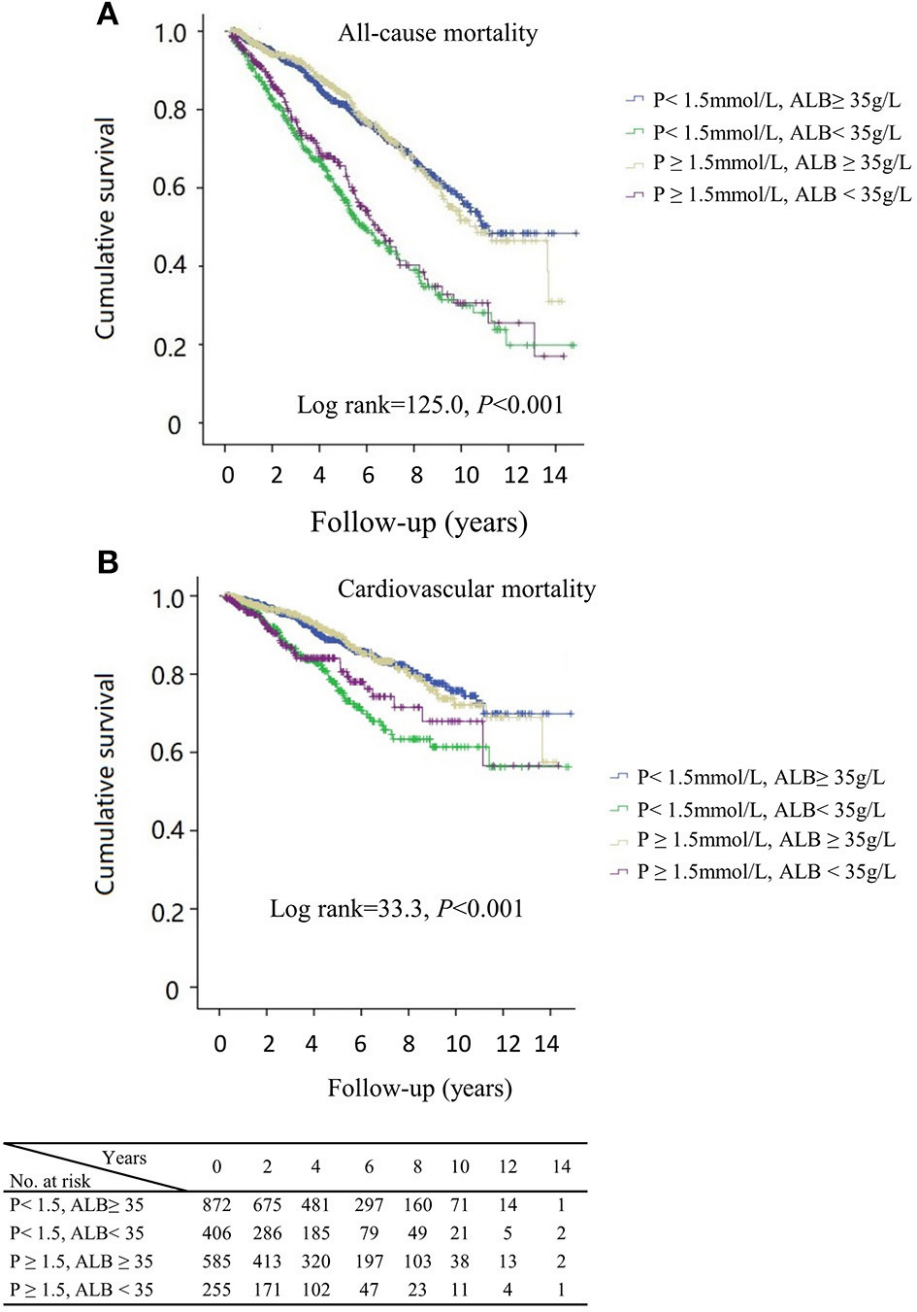
Kaplan–Meier curves of all-cause **(A)**, cardiovascular mortality **(B)** by subgroups of phosphorus and albumin levels, respectively. P, phosphorus; ALB, albumin.

**Figure 4:**
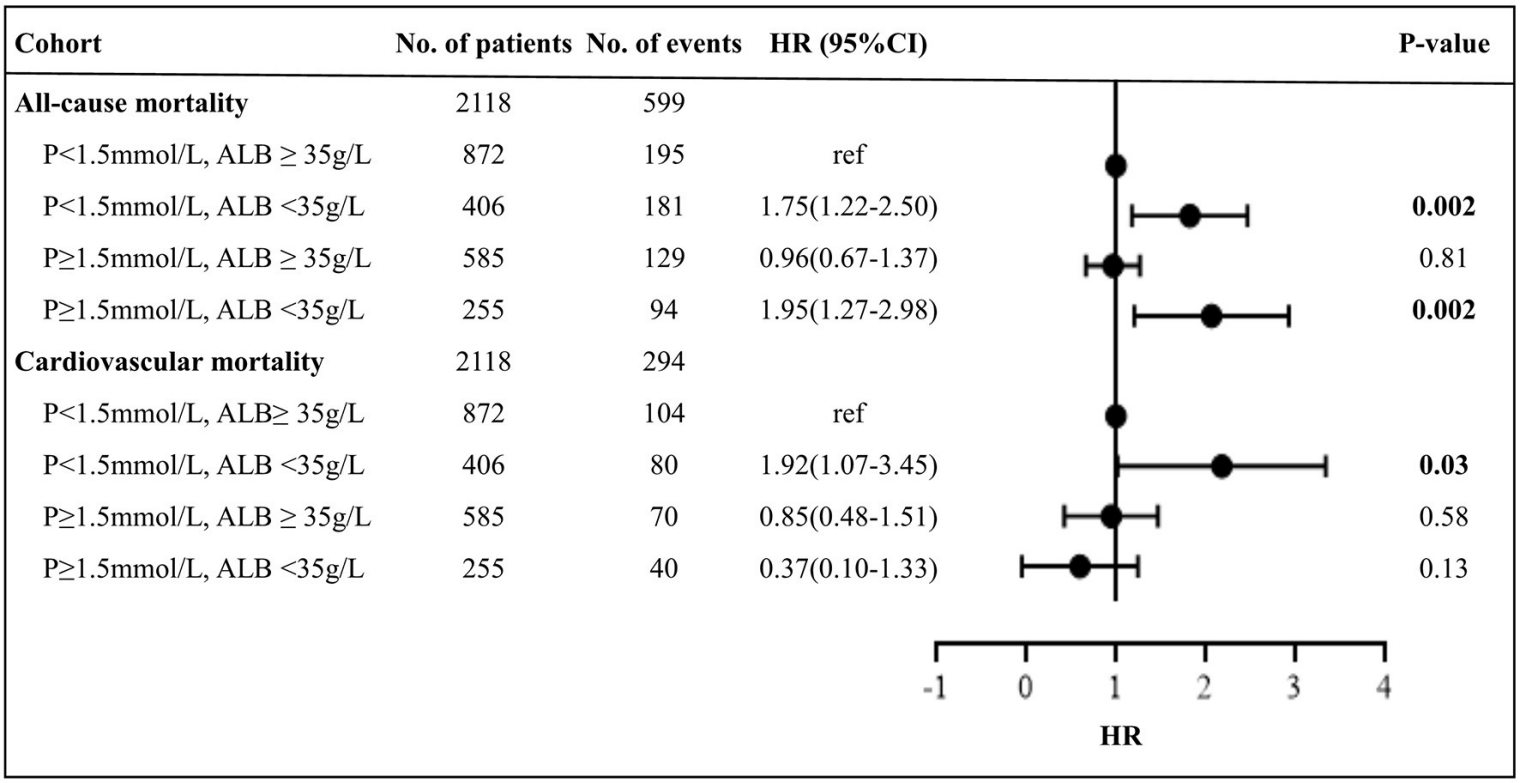
Forest plot of mortality outcomes by subgroups. P, phosphorus; ALB, albumin; HR, hazard ratio. Bold indicates significance at *p* < 0.05. Cox regression results was from model 4 which was adjusted by age, gender, and history of diabetes and cardiovascular disease, serum levels of hemoglobin, high-sensitive C-reactive protein, intact parathyroid hormone, systolic blood pressure, diastolic blood pressure, total cholesterol, triglyceride, low-density lipoprotein cholesterol, residual renal function, and uses of phosphate binders, calcitriol, and α-ketoacid. HR > 1 indicates patients with phosphorus <1.5 mmol/L and albumin ≥35 g/L is more beneficial, and <1 indicates patients with phosphorus and albumin levels in the respective group is more beneficial.

Figure 3 presents the survival curves for four categories of patients. The results showed higher all-cause and cardiovascular mortalities among patients with either concurrent low phosphorus and low albumin or high phosphorus and low albumin levels (*p* < 0.001). Notably, the cumulative incidences of both all-cause and cardiovascular mortality were significantly higher among patients with lower levels of serum albumin, irrespective of phosphorus values. The relative risk of mortality in relation to joint serum phosphorus/albumin levels is shown in Table 4 and Figure 4, with subjects in low phosphorus and high albumin group as a reference. After adjustment for potential confounders, including age, gender history of diabetes and cardiovascular disease, serum levels of hemoglobin, hs-CRP, iPTH, SBP, DBP, TC, TG, LDL-c, RRF, and uses of phosphate bidders, calcitriol, and α-ketoacid, patients with concurrent low phosphorus and low albumin levels had a 1.75-fold increase in all-cause mortality (95% CI 1.22–2.50, *p* = 0.002) and a 1.92-fold elevation in cardiovascular mortality (95% CI 1.07–3.45, *p* = 0.03). Adjusted hazard ratios of all-cause and cardiovascular mortality in patients with concurrent high phosphorus and low albumin levels were 1.95 (95%CI 1.27–2.98, *p* = 0.002) and 0.37 (95%CI 0.10–1.33, *p* = 0.13), respectively. However, no significantly increased risk of both all-cause and cardiovascular mortality was observed among patients with high phosphorus and high albumin relative to the reference group. These results implicated that correcting hyperphosphatemia at the “expense” of albumin would lead to increased mortality in patients undergoing CAPD.

**Table 4:** Analysis in associations between phosphorus and all-cause, cardiovascular mortality separately, and by albumin and phosphorus levels, respectively.

	**Model 1**		**Model 2**		**Model 3**		**Model 4**	
	**HR (95% CI)**	***P***	**HR (95% CI)**	***P***	**HR (95% CI)**	***P***	**HR (95% CI)**	***P***
**All-cause mortality**								
*p* < 1.5, ALB ≥ 35	Ref		Ref		Ref		Ref	
*p* < 1.5, ALB <35	2.54 (2.07–3.10)	** <0.001**	1.76 (1.43–2.16)	** <0.001**	1.56 (1.26–1.92)	** <0.001**	1.75 (1.22–2.50)	**0.002**
*P* ≥ 1.5, ALB ≥ 35	1.01 (0.81–1.26)	0.96	1.08 (0.86–1.35)	0.51	1.04 (0.83–1.30)	0.75	0.96 (0.67–1.37)	0.81
*P* ≥ 1.5, ALB <35	2.26 (1.77–2.89)	** <0.001**	2.03 (1.59–2.61)	** <0.001**	1.84 (1.43–2.36)	** <0.001**	1.95 (1.27–2.98)	**0.002**
**Cardiovascular mortality**								
*p* < 1.5, ALB ≥ 35	Ref		Ref		Ref		Ref	
*p* < 1.5, ALB <35	2.07 (1.55–2.77)	** <0.001**	1.41 (1.05–1.90)	**0.02**	1.22 (0.91–1.67)	0.17	1.92 (1.07–3.45)	**0.03**
*P* ≥ 1.5, ALB ≥ 35	1.02 (0.76–1.39)	0.88	1.12 (0.82–1.52)	0.47	1.08 (0.79–1.46)	0.64	0.85 (0.48–1.51)	0.58
*P* ≥ 1.5, ALB <35	1.78 (1.24–2.57)	**0.002**	1.57 (1.08–2.25)	**0.02**	1.40 (0.97–2.02)	0.07	0.37 (0.10–1.33)	0.13

*P, phosphorus; ALB, albumin. The unit for phosphorus and albumin was mmol/L and g/L, respectively. HR, hazard ratio.*

*Node at 1.5 mmol/L for phosphorus and 35 g/L for albumin was noted in restricted cubic splines and piecewise-linear model. Thereby phosphorus and albumin were categorized into groups as follows: (1) Phosphorus < 1.5 mmol/L, albumin ≥ 35 g/L, as the referenced group; (2) phosphorus < 1.5 mmol/L, albumin < 35 g/L; (3) phosphorus ≥ 1.5 mmol/L, albumin < 35 g/L; (4) phosphorus ≥ 1.5 mmol/L, albumin ≥ 35 g/L.*

*Model 1 was unadjusted.*

*Model 2 was adjusted for: age and gender.*

*Model 3 adjusted: model 2 + history of diabetes and cardiovascular disease.*

*Model 4 adjusted: model 3 + serum levels of hemoglobin, high-sensitive C-reactive protein, intact parathyroid hormone, systolic blood pressure, diastolic blood pressure, total cholesterol, triglyceride, low-density lipoprotein cholesterol, residual renal function, and uses of phosphate binders, calcitriol, and α-ketoacid.*

^*^Bold indicates significance at P < 0.05.

## Discussion

In the present study, we found that there were interactions between serum phosphorus and albumin in relation to all-cause and cardiovascular mortality. The association between phosphorus concentration and mortality was modified by albumin level, and high phosphorus with high albumin blunted the effects of the high phosphorus on all-cause and cardiovascular mortality.

Previous studies have indicated that patients who underwent dialysis have high prevalence of hyperphosphatemia, which is one of the most important risk factors associated with poor outcomes (7, 11). Correcting high serum phosphorus may ameliorate the adverse effects of high serum phosphorus on mortality in patients on dialysis. The reduction of dietary protein intake is widely recognized as one of cost-effective means for lowering serum phosphorus level in patients with chronic kidney disease (13, 29). However, phosphorus and protein intake is closely related, and it is reasonable to consider that enhanced dietary protein intake would increase both serum phosphorus and serum albumin levels, whereas insufficiency of protein intake may result in lower serum phosphorus concentration and a concurrent decline in serum albumin (30).

Our data corroborated previous findings that hypoalbuminemia was a significant determinant of all-cause and cardiovascular mortality (19, 22), but no such correlation was found for serum phosphorus when assessed as a continuous variable. However, non-linearities were evident separately for phosphorus and albumin in their relations to mortality. Notably, there were significant interactions between phosphorus and albumin in their associations with both all-cause and cardiovascular mortality. Our finding was consistent with the previous study that the associations of serum phosphorus and albumin concentrations with mortality are modified by each other over time in a cohort study of patients on incident HD and PD (14). Therefore, combination effects of serum albumin and phosphorus should be evaluated concurrently with respect to clinical outcomes in patients on incident dialysis (20).

It has been demonstrated that both hyperphosphatemia and hypoalbuminemia are independent risk factors for mortality in patients undergoing dialysis (1, 5, 7, 21, 22). But there are several studies showing no relation between serum phosphorus and survival in patients undergoing pre-dialysis and dialysis (31–33). We found that patients with high phosphorus and low albumin concentrations (phosphorus ≥1.5 mmol/L and albumin < 35 g/L) had the highest risk of all-cause mortality across the four groups, compared to those with low serum phosphorus and high albumin. On the contrary, high phosphorus with albumin level higher than 35 g/L was not associated with an increased risk of all-cause and cardiovascular mortality in patients undergoing CAPD. Our data suggested that associations between high phosphorus and all-cause mortality were indeed modified by serum albumin concentration, which is in line with previous studies that demonstrated a worse outcome among patients with higher phosphorus and lower albumin (16, 20).

The K/DOQI and KDIGO guidelines suggest the three cornerstone approaches to dispose hyperphosphatemia (13, 29). As a first-line approach, dietary phosphorus control, by restricting phosphorus-rich foods, avoiding phosphorus additives in processed foods, and using wet cooking methods and substitute foods with relative lower phosphorus bioavailability (16, 20), along with maintaining adequate dietary protein intake are essential in the management of hyperphosphatemia and prevent protein-energy wasting in patients on dialysis. In a study of 884 patients undergoing incident PD, a diet with a higher plant-based protein–total protein ratio is found to be related to lower all-cause and cardiovascular mortality, especially in those without hypoalbuminemia (34). On the other hand, restricting dietary protein, in order to obtain a neutral phosphorus balance, has been demonstrated to impose the risk of hypoalbuminemia and protein-energy wasting (15, 30). Previous study revealed that patients undergoing HD with a decrease in serum phosphorus and a concomitant reduction in normalized protein nitrogen appearance, a surrogate of dietary protein intake, had a 11% higher risk of mortality than those with an increase in both parameters over 6 months (16). Furthermore, a *post-hoc* analysis from the Hemodialysis (HEMO) study showed that more restrictive prescribed dietary phosphate was associated with poorer indices of nutritional status and did not confer a survival advantage among patients on prevalent HD. Instead, a more liberal phosphate prescription was related to increased survival (14). Consistently, our results showed that patients with low concurrent levels of serum phosphorus and albumin (phosphorus < 1.5 mmol/L and albumin < 35 g/L) had an increased risk of both all-cause and cardiovascular mortality. In contrast, the risk of mortality did not differ in high serum albumin categories, irrespective of phosphorus levels. These data suggest that interventions to control serum phosphorus along with reduced albumin overweight the benefit of lower serum phosphorus and cause greater mortality. In addition, there have been studies showing that low serum phosphorus was associated with higher all-cause and cardiovascular mortality risks (35, 36). In our present study, low phosphorus showed a non-significant trend toward increased mortality, probably due to relatively small sample size, which might have obscured its potential effect on survival. Therefore, maintaining adequate dietary protein intake is also one of important strategies in the management of hyperphosphatemia (14).

The control of serum phosphorus by higher dose PD is made difficult due to time-consuming, burdensome, and out-of-pocket costs for patients (29). Meanwhile, higher dose PD has been associated with better phosphorus control (37), but probably worse nutrition status (37–40) and equivalent outcomes (41). Therefore, phosphate binders might be appropriate for patients undergoing CAPD to maintain acceptable serum phosphorus levels and avoid the risk of malnutrition in clinical practice. In the most recent KDIGO and K/DOQI guidelines, non-calcium-based phosphorus binders are recommended to use as first-line phosphorus binders (13, 29). Available non-calcium-based phosphorus binders, such as lanthanum carbonate and sevelamer carbonate, are shown to reduce serum phosphorus levels and maintain serum albumin (42, 43). Recently, novel iron-based phosphorus binders with a low pill burden have demonstrated efficacy at decreasing serum phosphorus. More importantly, it has been shown to increase serum albumin levels among patients with hypoalbuminemia, possibly by allowing patients to increase their dietary intake of protein (44–46). In our cohort study, 849 subjects (40%) received phosphorus binders, association of serum phosphorus with mortality risk was adjusted for use of phosphorus binders. However, we did not assess whether there was difference between patients prescribed and those not prescribed phosphorus binders.

The strengths of the study included prospective study design with a large incident PD cohort in a single center. This study explored serum phosphorus–albumin interaction and associations with mortality among CAPD population for the first time to our knowledge. However, there are several limitations in our studies. First, we only enrolled patients for CAPD, and our findings may therefore not be representative of those treated with automated PD or HD due to the difference in excretion mechanism and amount of excreted phosphorus. Second, the analysis was based on baseline data rather than changes of these parameters. Third, we cannot eliminate the potential confounding impact related to clinical outcomes, although we tried to adjust for many relevant confounders in the analysis.

## Conclusions

Serum albumin modified the association between phosphorus level and mortality, with high concurrent levels of serum phosphorus and albumin not associated with mortality. Lower albumin concentration was related to an increased risk of all-cause and cardiovascular mortality independent of phosphorus levels. The findings implicate that controlling serum phosphorus without reducing albumin could improve clinical outcomes in patients undergoing CAPD.

## Data Availability Statement

The original contributions presented in the study are included in the article/supplementary material, further inquiries can be directed to the corresponding author/s.

## Ethics Statement

The studies involving human participants were reviewed and approved by Clinical Research Ethics Committee of the First Affiliated Hospital of Sun Yat-sen University. The patients/participants provided their written informed consent to participate in this study.

## Author Contributions

HM and XY developed and designed the research. NH and HL conducted the research and wrote the paper. NH, LF, QZ, DF, and LG analyzed the data. CY provided essential materials for the research. HM had primary responsibility for the final content. All authors have read and approved the final manuscript.

## Funding

This work was partially supported by the Guangzhou Science and technology plan (Grant no. 201807010002); the Natural Science Foundation of Guangdong Province, China (Grant no. 2017A030310199); National Natural Science Foundation of China (Grant no. 81700583), 5010 Clinical Program of Sun Yat-sen University (2017007); the National Key Research and Development Project (Grant no. 2016YFC0906101); the Operational Grant of Guangdong Provincial Key Laboratory (Grant no. 2017B030314019); and the Key Laboratory of National Health Commission and Key Laboratory of Nephrology, Guangdong Province, Guangzhou, China (Grant no. 2002B60118).

## Conflict of Interest

The authors declare that the research was conducted in the absence of any commercial or financial relationships that could be construed as a potential conflictof interest.

## Publisher's Note

All claims expressed in this article are solely those of the authors and do not necessarily represent those of their affiliated organizations, or those of the publisher, the editors and the reviewers. Any product that may be evaluated in this article, or claim that may be made by its manufacturer, is not guaranteed or endorsed by the publisher.
